# 1,3-Dichlorbenzol – Evaluierung eines Biologischen Leitwertes (BLW): Beurteilungswerte in biologischem Material

**DOI:** 10.34865/bb54173d11_1or

**Published:** 2026-03-30

**Authors:** Andrea Kaifie, Wobbeke Weistenhöfer, Thomas Kraus, Hans Drexler, Andrea Hartwig, MAK Commission

**Affiliations:** 1 Friedrich-Alexander-Universität Erlangen-Nürnberg, Institut und Poliklinik für Arbeits-, Sozial- und Umweltmedizin Henkestraße 9–11 91054 Erlangen Deutschland; 2 Institut für Arbeits-, Sozial- und Umweltmedizin, Uniklinik RWTH Pauwelsstraße 30 52074 Aachen Deutschland; 3 Institut für Angewandte Biowissenschaften, Abteilung Lebensmittelchemie und Toxikologie, Karlsruher Institut für Technologie (KIT) Adenauerring 20a, Geb. 50.41 76131 Karlsruhe Deutschland; 4 Ständige Senatskommission zur Prüfung gesundheitsschädlicher Arbeitsstoffe, Deutsche Forschungsgemeinschaft, Kennedyallee 40, 53175 Bonn, Deutschland. Weitere Informationen: Ständige Senatskommission zur Prüfung gesundheitsschädlicher Arbeitsstoffe | DFG

**Keywords:** 1,3-Dichlorbenzol, 3,5-Dichlorkatechol, Biologischer Leitwert, BLW, Urin, 13673-92-2, urine, 13673-92-2

## Abstract

In 2025, the German Commission for the Investigation of Health Hazards of Chemical Compounds in the Work Area has evaluated a biological guidance value (BLW) for 1,3‑dichlorobenzene [541-73-1]. Relevant publications were identified from a literature search. For the derivation of the BLW, an experimental study was available that demonstrated a good correlation between the concentration of 1,3-dichlorobenzene in the air and 1,3-dichlorobenzene in blood as well as the urinary metabolites 3,5‑dichlorocatechol, 2,4‑dichlorophenol and 3,5-dichlorophenol. The data of the experimental study conducted with resting volunteers for 6-hours were extrapolated to account for an exposure of 8 hours and an increased respiratory volume at the work place. At the level of the MAK value of 2 ml/m^3^, an average value of 40 mg 3,5-dichlorocatechol (after hydrolysis)/g creatinine can be expected and was set as BLW. Sampling time is at the end of the shift, after several previous shifts.

**Table:** 

**BLW (2025)**	**40 mg 3,5-Dichlorkatechol (nach Hydrolyse)/g Kreatinin**
	Probenahmezeitpunkt: am Schichtende, bei Langzeitexposition nach mehreren vorangegangenen Schichten
	
Synonyma	*m*-Dichlorbenzol
Formel	C_6_H_4_Cl_2_
Molmasse	147 g/mol (IFA 2026)
Schmelzpunkt	–22 °C (IFA 2026)
Siedepunkt	173 °C (IFA 2026)
Dampfdruck bei 20 °C	2,4 hPa (IFA 2026)
Dichte bei 20 °C	1,29 g/cm^3^ (IFA 2026)
log K_OW_	3,52 (IFA 2026)
Wasserlöslichkeit bei 60 °C	0,201 g/l (IFA 2026)
	
**MAK-Wert (2007)**	**2 ml/m^3^ ≙ 12 mg/m^3^**
Hautresorption	–
Krebserzeugende Wirkung	–
Fruchtschädigende Wirkung	Gruppe C

1,3-Dichlorbenzol ist eine farblose Flüssigkeit mit einem markanten Geruch, die als Abbauprodukt bei der Herstellung von Silikonkautschuk entsteht, wenn Bis(2,4-dichlorbenzoyl)peroxid (2,4-DCBP) als Radikalstarter eingesetzt wird (Herkert et al. 2018).

## Metabolismus und Toxikokinetik

**Abb. 1:**
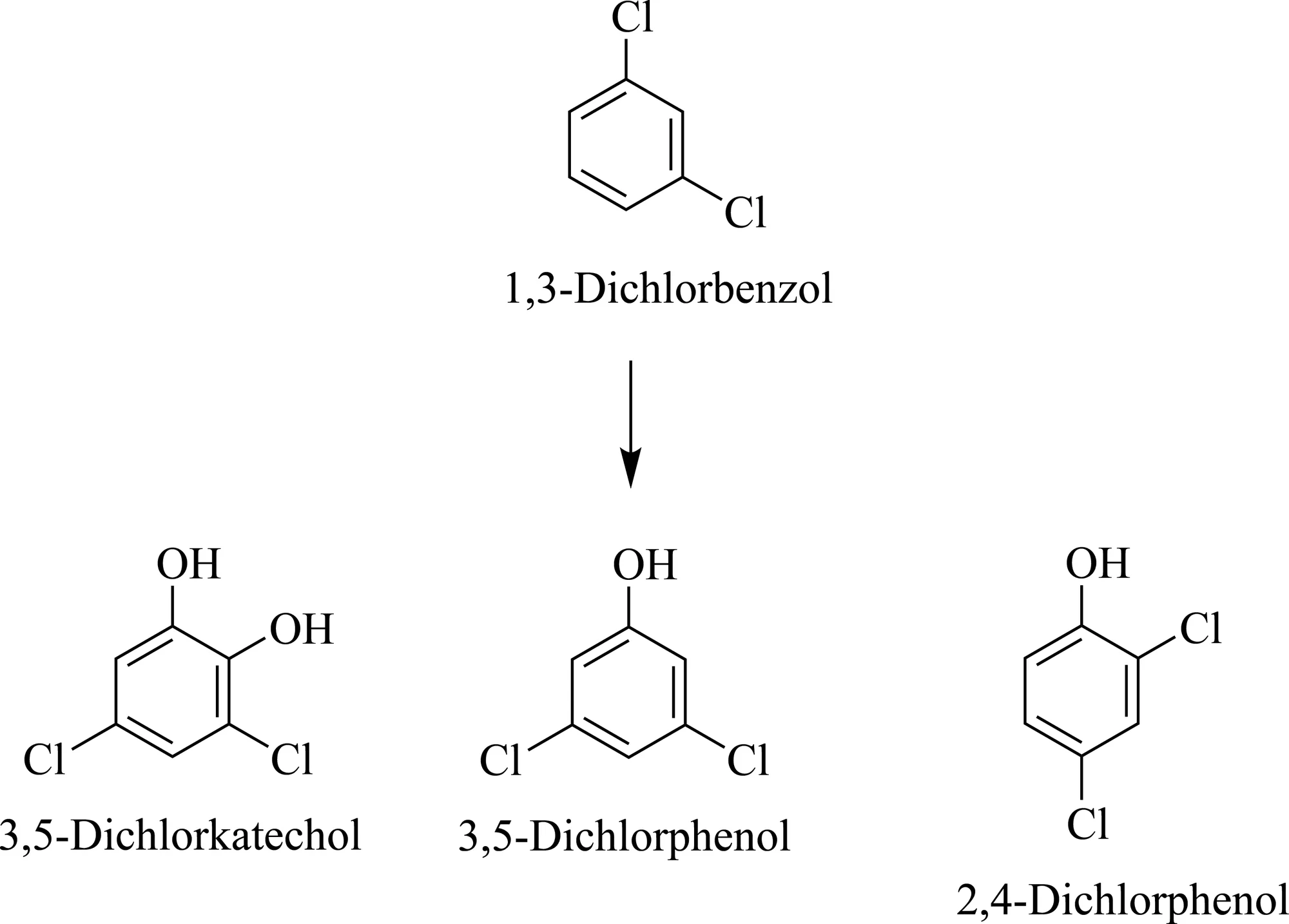
Phenolische Metaboliten des 1,3-Dichlorbenzol im Urin beim Menschen

1,3-Dichlorbenzol wird im Körper u. a. zu den Metaboliten 3,5-Dichlorkatechol, 3,5-Dichlorphenol und 2,4-Dichlorphenol verstoffwechselt (Abbildung 1), die über den Urin als Glucuronid und Sulfat ausgeschieden werden.

Schettgen et al. (2022) untersuchten die innere Belastung mit 1,3-Dichlorbenzol und bestimmten die Metaboliten 3,5-Dichlorkatechol, 2,4-Dichlorphenol und 3,5-Dichlorphenol im Urin bei 104 Beschäftigten (82 Männer und 22 Frauen) einer Firma, die Silikonkautschuk herstellt und 2,4-DCBP als Radikalstarter einsetzt. In mehr als 89 % aller Urinproben wurden die drei Metaboliten detektiert. Die maximalen Konzentrationen betrugen 26,9 mg 3,5-Dichlorkatechol/l, 7,68 mg 2,4-Dichlorphenol/l und 0,39 mg 3,5-Dichlorphenol/l Urin.

## Kritische Toxizität

Es liegen keine Daten zur Toxizität beim Menschen vor.

Empfindlichster Effekt des 1,3-Dichlorbenzols ist die Induktion fremdstoffmetabolisierender Enzyme, u. a. der Glucuronosyltransferase I in einer 28-Tage-Studie mit Schlundsondenapplikation an Ratten, in der ein LOEL (lowest observed effect level) von 4 mg/kg KG und Tag erhalten wurde (Hoechst AG 1989). Es ist davon auszugehen, dass bei diesem besonders sensiblen Endpunkt die kurzfristig hohe Belastung durch die Bolusgabe die Höhe des LOEL mitbestimmt hat. Die Induktion der Leberenzyme muss als Auslöser der Veränderungen in den Schilddrüsenfollikeln der Ratten angesehen werden, die in einer 90-Tage-Studie ab 37 mg 1,3-Dichlorbenzol/kg KG und Tag (LOAEL: lowest observed adverse effect level) signifikant vermehrt auftraten (McCauley et al. 1995). Die beobachteten Schilddrüsenveränderungen sind auch für den Menschen relevant, treten aber erst bei höheren Dosierungen als bei der Ratte auf (Lewandowski et al. 2004), da dem Menschen durch die Fähigkeit, Schilddrüsenhormone zu speichern, bessere Schutzmechanismen zur Verfügung stehen. In der 90-Tage-Studie an der Ratte traten bei 9 mg/kg KG keine adversen Wirkungen an der Schilddrüse auf. Aus diesem NOAEL (no observed adverse effect level) von der Ratte wurde ein MAK-Wert von 2 ml 1,3-Dichlorbenzol/m^3^ (12 mg/m^3^) abgeleitet (Greim 2008). Bei einer Reevaluierung wurde mit einer genaueren Speziesübertragung eine entsprechende Luftkonzentration am Arbeitsplatz von 18 mg/m^3^ errechnet und der MAK-Wert bestätigt (Hartwig 2012), da der zugrunde liegende NOAEL für Schilddrüsenepithelveränderungen an Ratten gewonnen wurde und Ratten in Bezug auf Schilddrüsenwirkungen als empfindlicher als der Mensch gelten (Greim 2008; Hartwig 2012).

## Belastung und Beanspruchung

In einer Arbeitsplatzsimulationsanlage erfolgte die Exposition 10 gesunder männlicher Probanden gegen 1,3-Dichlorbenzol über insgesamt sechs Stunden mit anschließendem Biomonitoring von 1,3‑Dichlorbenzol im Blut und Bestimmung der Metaboliten 3,5-Dichlorkatechol (DCK), 2,4‑Dichlorphenol (DCP) und 3,5-Dichlorphenol im Urin (Schettgen et al. 2023). Dabei gab es drei Expositionsszenarien: 1,5 ml 1,3-Dichlorbenzol/m^3^ (8 mg/m^3^), 0,7 ml/m^3^ (4 mg/m^3^) und 1,5 ml/m^3^ mit Tragen einer Atemschutzmaske für Gase und Dämpfe (3M, 4251+). Während der Exposition erfolgte keine körperliche Belastung. Beanspruchungsparameter wurden nicht untersucht.

### 1,3-Dichlorbenzol im Blut

**Abb. 2:**
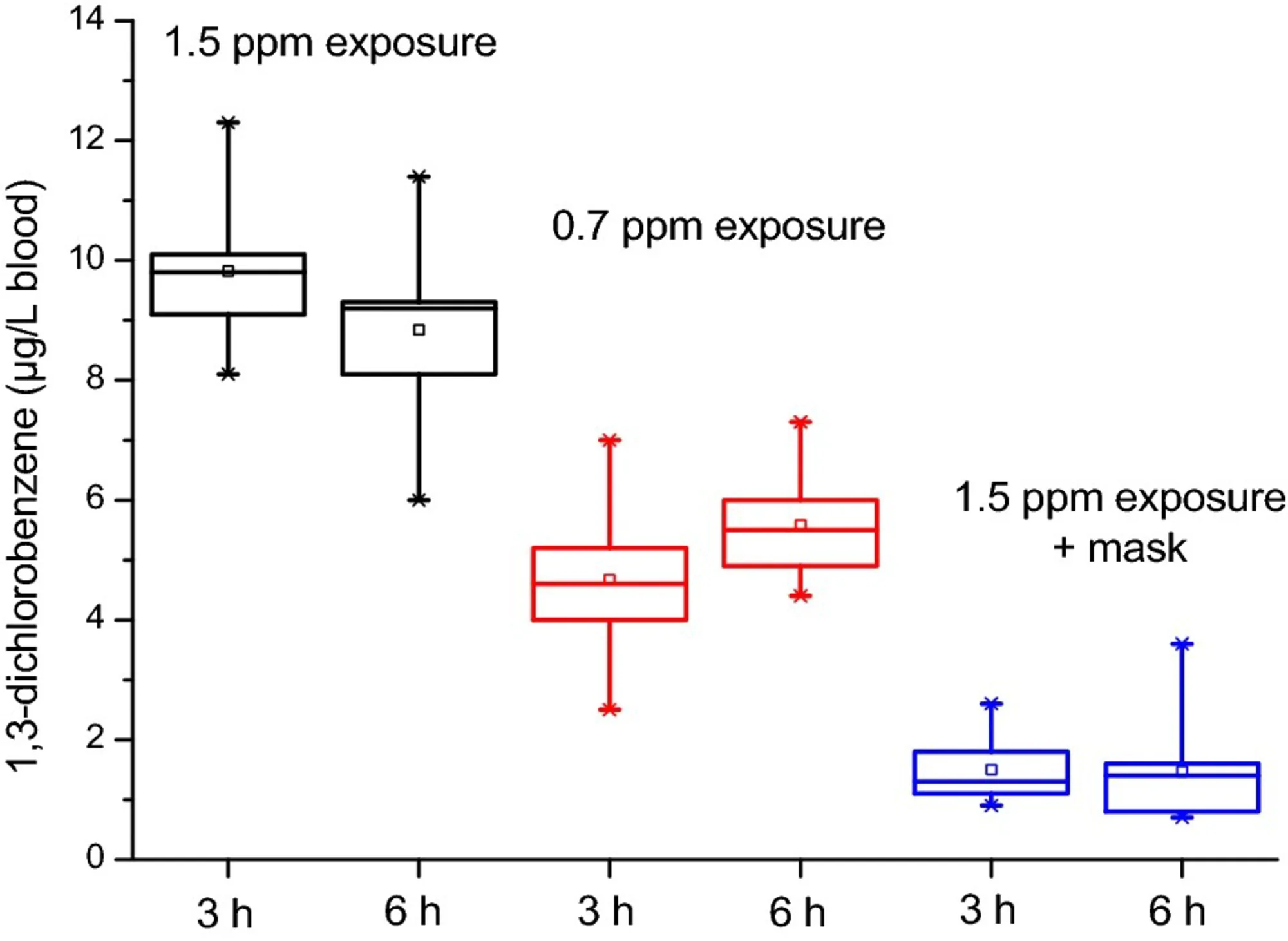
Konzentration von 1,3-Dichlorbenzol im Blut nach 3 und 6 Stunden in unterschiedlichen Expositionsszenarien (aus Schettgen et al. 2023, publiziert unter der CC BY 4.0-Lizenz bei Springer Nature)

Die Blutentnahmen zur Bestimmung von 1,3-Dichlorbenzol erfolgten drei Stunden nach Expositionsbeginn und nach sechs Stunden am Ende der Exposition. Die 1,3‑Dichlorbenzolkonzentration im Blut unterschied sich hierbei nicht signifikant zwischen den Beprobungszeitpunkten. Daraus lässt sich schließen, dass das Fließgleichgewicht nach etwa drei Stunden erreicht wurde. Es wurden bei Exposition gegen 1,5 ml 1,3-Dichlorbenzol/m^3^ im Median 1,3-Dichlorbenzol-Konzentrationen von 9,7 µg/l Blut (nach drei Stunden) und 9,0 µg/l Blut (nach sechs Stunden) bestimmt (Abbildung 2).

Für den Zusammenhang zwischen 1,3-Dichlorbenzol in der Luft und 1,3-Dichlorbenzol im Blut wurde folgende Regressionsgleichung (1) abgeleitet:

1,3-Dichlorbenzol [μg/l Blut] = 5,26 × 1,3-Dichlorbenzol [ml/m^3^ Luft] + 1,45 (1)

Daraus leitet sich für eine Exposition in Höhe des MAK-Wertes von 2 ml 1,3‑Dichlorbenzol/m^3^ eine Konzentration von 12 µg 1,3‑Dichlorbenzol/l Blut ab.

### Metaboliten im Urin

Urinproben wurden vor Exposition (t_0_) und über 24 Stunden gesammelt. 3,5-Dichlorkatechol zeigte die höchste Konzentration, gefolgt von 2,4‑Dichlorphenol und 3,5-Dichlorphenol. Unter einer Exposition gegen 1,5 ml 1,3‑Dichlorbenzol/m^3^ war die maximale Urinkonzentration für 3,5-Dichlorkatechol (11963 µg/g Kreatinin) nach im Mittel 6,6 Stunden erreicht. Die Maximalwerte für 2,4-Dichlorphenol (3515 µg/g Kreatinin) und 3,5-Dichlorphenol (173 µg/g Kreatinin) wurden nach 6,4 Stunden gemessen. Die Halbwertszeiten für die Metaboliten lagen bei 9,7 Stunden für 3,5-Dichlorkatechol, 5,8 Stunden für 2,4-Dichlorphenol und 4,8 Stunden für 3,5-Dichlorphenol.

Es wurden für eine Exposition über 6 Stunden folgende Regressionsgleichungen (2–4) für den Zusammenhang zwischen 1,3-Dichlorbenzol in der Luft und 3,5-Dichlorkatechol, 2,4-Dichlorphenol und 3,5-Dichlorphenol im Urin abgeleitet:

3,5-Dichlorkatechol [μg/g Kreatinin] = 8472 × 1,3-Dichlorbenzol [ml/m^3^ Luft] –744 (2)

2,4-Dichlorphenol [μg/g Kreatinin] = 2452 × 1,3-Dichlorbenzol [ml/m^3^ Luft] –163 (3)

3,5-Dichlorphenol [μg/g Kreatinin] = 128,4 × 1,3-Dichlorbenzol [ml/m^3^ Luft] –20 (4)

Extrapoliert auf eine Expositionszeit von 8 Stunden wurden von Schettgen et al. (2023) für eine Exposition in Höhe des MAK-Wertes von 2 ml 1,3-Dichlorbenzol/m^3^ folgende Metabolitenkonzentrationen im Urin (nach Hydrolyse) angegeben: 3,5-Dichlorkatechol: 21 600 μg/g Kreatinin, 2,4-Dichlorphenol: 6300 μg/g Kreatinin und 3,5-Dichlorphenol: 320 μg/g Kreatinin.

Literbezogene Konzentrationen wiesen eine höhere Variabilität auf (r = 0,6 und kleiner) als kreatininbezogene. Bei den Probanden lag ein Bereich von 0,12–5,59 g Kreatinin/l Urin vor, der somit zum Teil außerhalb der empfohlenen Kreatininkonzentrationen von 0,3–3 g Kreatinin/l Urin lag. Die Werte wurden trotzdem in die Analyse eingeschlossen (n = 38 von 203 Urinproben, ca. 19 %). Ein Proband gab über 24 Stunden jeweils nur drei Urinproben ab.

## Auswahl der Indikatoren

Ein Biomonitoring 1,3-Dichlorbenzol-exponierter Personen kann über die Bestimmung der Konzentration von 1,3-Dichlorbenzol im Vollblut erfolgen.

Als Metaboliten des 1,3-Dichlorbenzols im Urin stehen zur Verfügung: 3,5-Dichlorkatechol, 2,4‑Dichlorphenol, 3,5-Dichlorphenol sowie die zwei isomeren 2,4‑Dichlorphenylmercaptursäure und 3,5-Dichlorphenylmercaptursäure.

## Untersuchungsmethoden

Zur Bestimmung von 1,3-Dichlorbenzol im Vollblut wird eine Headspace-Gaschromatographie-Massenspektrometrie (GC/MS) und für die Metaboliten 3,5-Dichlorkatechol, 2,4-Dichlorphenol und 3,5-Dichlorphenol im Urin eine vollautomatisierte Festphasenextraktion (SPE) kombiniert mit einer Flüssigchromatographie-Tandem-Massenspektrometrie (LC-MS/MS) nach Hydrolyse als Untersuchungsmethode eingesetzt (Schettgen et al. 2023). Für die Bestimmung der Mercaptursäuren im Urin liegt ein online-SPE LC-MS/MS-Verfahren vor (Schettgen et al. 2024).

## Hintergrundbelastung

Es liegen keine Daten zur Hintergrundbelastung der Allgemeinbevölkerung im erwerbsfähigen Alter in Deutschland mit 1,3-Dichlorbenzol oder dessen Metaboliten vor.

In einer repräsentativen Stichprobe (Altersgruppe ≥ 18 Jahre) der Allgemeinbevölkerung in den USA (National Health and Nutrition Examination Survey (NHANES)) befand sich im Survey-Zeitraum 2015/2016 der Referenzwert unterhalb der Nachweisgrenze von 0,025 ng 1,3-Dichlorbenzol/ml Blut (CDC 2025).

## Evaluierung eines Biologischen Leitwertes (BLW)

Für die Evaluierung eines Grenzwertes liegt nur die Studie von Schettgen et al. (2023) vor. Dabei sind die folgenden Limitationen der Studie zu berücksichtigen:

- Es erfolgte während der Exposition gegen 1,3-Dichlorbenzol keine körperliche Belastung. Bei einem Blut:Luft-Verteilungskoeffizienten von 87,6 für das chemisch ähnliche 1,4-Dichlorbenzol wird daher für die Berücksichtigung des erhöhten Atemvolumens am Arbeitsplatz der Faktor 2 in die Berechnung des Grenzwertes einbezogen.
- Die Studie erfolgte an lediglich 10 Personen.
- Bei fast 20 % der Probanden lag die Kreatininkonzentration im Urin außerhalb des empfohlenen Bereichs von 0,3–3 g/l Urin (WHO 1996), so dass die Aussagekraft dieser Urinproben eingeschränkt ist.
- Die Exposition erfolgte über 6 statt 8 Stunden mit einer halbstündigen Pause (2 × 3 Stunden).
- Die Expositionshöhe lag mit 1,5 ml 1,3-Dichlorbenzol/m^3^ unterhalb des MAK-Wertes von 2 ml 1,3‑Dichlorbenzol/m^3^.

Aufgrund der genannten Unsicherheiten wird ein BLW für den Metaboliten 3,5-Dichlorkatechol im Urin, der die höchste Konzentration der Parameter aufwies, festgelegt. Unter Berücksichtigung eines Faktors von 2 für das erhöhte Atemvolumen am Arbeitsplatz und einer Extrapolation von 6 auf 8 Stunden Exposition wird in Korrelation zum MAK-Wert von 2 ml 1,3‑Dichlorbenzol/m^3^

**ein BLW von 40 mg 3,5-Dichlorkatechol (nach Hydrolyse)/g Kreatinin abgeleitet.**

Da es aufgrund der Halbwertszeit von 3,5-Dichlorkatechol von 9,7 Stunden über mehrere Schichten zu einer Kumulation der Metaboliten im Urin kommen kann, sollte die Probenahme am Schichtende, bei Langzeitexposition nach mehreren vorangegangenen Schichten erfolgen.

## Interpretation

Der BLW für 3,5-Dichlorkatechol bezieht sich auf normal konzentrierten Urin, bei dem der Kreatiningehalt im Bereich von 0,3–3 g/l Urin liegt. In der Regel empfiehlt sich bei Urinproben außerhalb der oben genannten Grenzen die Wiederholung der Messung beim normal hydrierten Probanden (Bader und Ochsmann 2010).
